# Case report: Rare genetic liver disease - a case of congenital hepatic fibrosis in adults with autosomal dominant polycystic kidney disease

**DOI:** 10.3389/fmed.2024.1344151

**Published:** 2024-02-07

**Authors:** Ying Liu, Ping Zhu, Jiajun Tian

**Affiliations:** ^1^Department of Gastroenterology and Hepatology, Tianjin Third Central Hospital, Tianjin, China; ^2^Tianjin Key Laboratory of Extracorporeal Life Support for Critical Diseases, Tianjin, China; ^3^Artificial Cell Engineering Technology Research Center, Tianjin Institute of Hepatobiliary Disease, Tianjin, China

**Keywords:** PKD1 gene mutation, congenital hepatic fibrosis, ADPKD, adult female, case report

## Abstract

Congenital hepatic fibrosis (CHF) is considered to be a rare autosomal recessive hereditary fibrocystic liver disease, mainly found in children. However, cases of adult CHF with autosomal dominant polycystic kidney disease (ADPKD) caused by PKD1 gene mutation are extremely rare. We report a 31-year-old female patient admitted for esophageal and gastric variceal bleeding. Physical examination revealed significant splenomegaly, biochemical tests showed a slight increase in liver enzymes, and a decrease in platelet count. Imaging examinations showed significant dilatation of the common bile duct and intrahepatic bile ducts, as well as multiple renal cysts. Liver biopsy revealed enlarged portal areas, bridging fibrosis, and numerous variably shaped small bile ducts. Genetic testing identified two unique mutations in the PKD1 gene, identified as biallelic mutations compound heterozygous mutations composed of a mutation inherited from the father (c.8296 T > C) and one from the mother (c.9653G > C). Based on multiple test results, the patient was diagnosed with the portal hypertension type CHF associated with ADPKD. During her initial hospital stay, the patient underwent endoscopic treatment for gastrointestinal bleeding. To date, the patient has recovered well. Moreover, a significant reduction in varices was observed in a gastroscopy examination 18 months later.

## Introduction

In 1961, Kerr (1) first introduced the term “Congenital hepatic fibrosis (CHF)” to describe a unique liver fibrosis condition distinct from cirrhosis. CHF is a rare inherited disorder that typically presents in childhood or adolescence. It is characterized by hepatosplenomegaly and portal hypertension. Interestingly, liver function may remain unaltered or exhibit only minor abnormalities (2). CHF can present as isolated hepatic involvement, but it often co-occurs with Caroli’s syndrome or polycystic kidney disease (PKD) (3). Autosomal recessive polycystic kidney disease (ARPKD) and autosomal dominant polycystic kidney disease (ADPKD) are the most prevalent forms of PKD. ARPKD is inherited in an autosomal recessive pattern, usually due to mutations in the PKHD1 gene, while ADPKD is inherited in an autosomal dominant pattern, often caused by mutations in either the PKD1 or PKD2 genes. CHF can be observed in approximately 50% of ARPKD cases (4), whereas it is less common in ADPKD cases. The most prevalent hepatic manifestation of ADPKD is the formation of liver cysts. With advancements in molecular biology techniques, several cases of childhood CHF accompanied by ADPKD, caused by PKD1 gene mutations, have been reported (5–7). However, in the past two decades, there have been relatively fewer reports on adult CHF combined with ADPKD.

Here, we present a rare case of CHF with ADPKD in an adult female, caused by PKD1 variants. This diagnosis was confirmed through medical imaging, liver biopsy, and genetic testing. In addition to presenting the case, we analyze the computed tomography (CT) and magnetic resonance cholangiopancreatography (MRCP) imaging results, discussed the histopathological features, diagnosis, and treatment of CHF, and reviewed relevant literature.

## Case presentation

A 31-year-old adult female patient was admitted to our hospital, with a one-day history of massive hematemesis and melena. The patient denied past medical history. The patient’s 61-year-old mother is asymptomatic, while the patient’s father presents with hepatomegaly, splenomegaly, and bilateral multiple renal cysts. Her brother underwent a splenectomy five years ago due to bleeding from esophagogastric varices caused by portal hypertension and also has a history of bilateral multiple renal cysts. Physical examination showed that the patient has pale conjunctiva and an enlarged spleen, with no other abnormalities. Laboratory examination revealed a hemoglobin level of 55 g/L (reference range: 115–150 g/L), and a platelet count of 115 × 109/L (reference range: 125–350 × 109/L). The liver biochemical profile showed elevated alkaline phosphatase of 309 U/L (reference range: 35–100 U/L) and γ glutamyl transpeptidase of 218 U/L (reference range: 7-45 U/L). Levels of white blood cell count, alanine aminotransferase, aspartate aminotransferase, bilirubin, creatinine and prothrombin time were within the normal range. Tests for hepatitis B surface antigen, hepatitis C virus, cytomegalo virus, Epstein–Barr virus antibodies, examination of serum ceruloplasmin, serum iron, transferrin saturation, smooth muscle actin antibodies, liver-kidney microsomal antibodies, anti-nuclear antibodies and immune-globulin (Ig) G and IgM were performed and yielded negative results.

**Figure 1:**
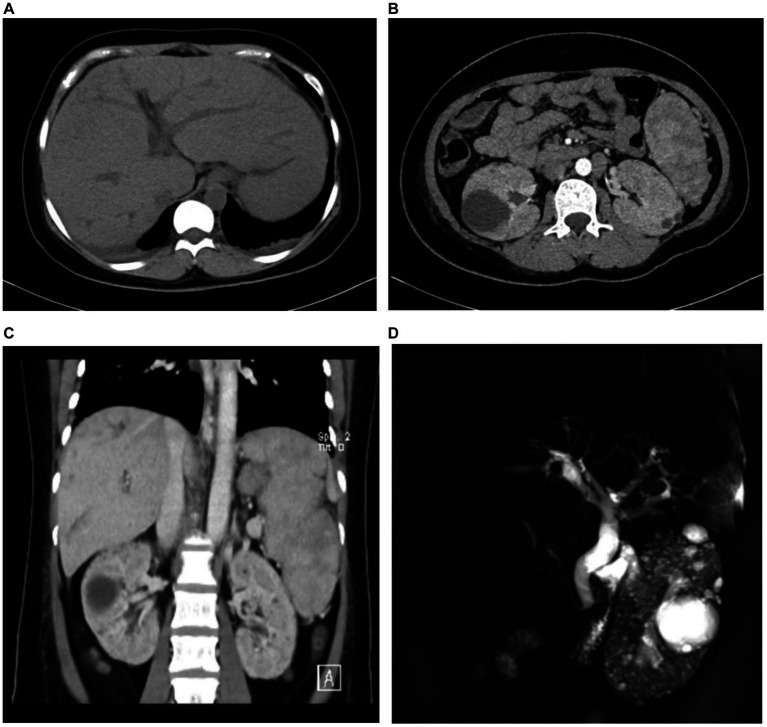
The medical imaging results of the patient. Abdominal contrast-enhanced CT revealed intrahepatic bile ducts dilation **(A)**, multiple renal cysts **(B)**, and splenomegaly **(C)**. Magnetic resonance cholangiopancreatography (MRCP) scan showed the dilation of both extrahepatic and intrahepatic bile ducts, as well as multiple renal cysts **(D)**.

**Figure 2:**
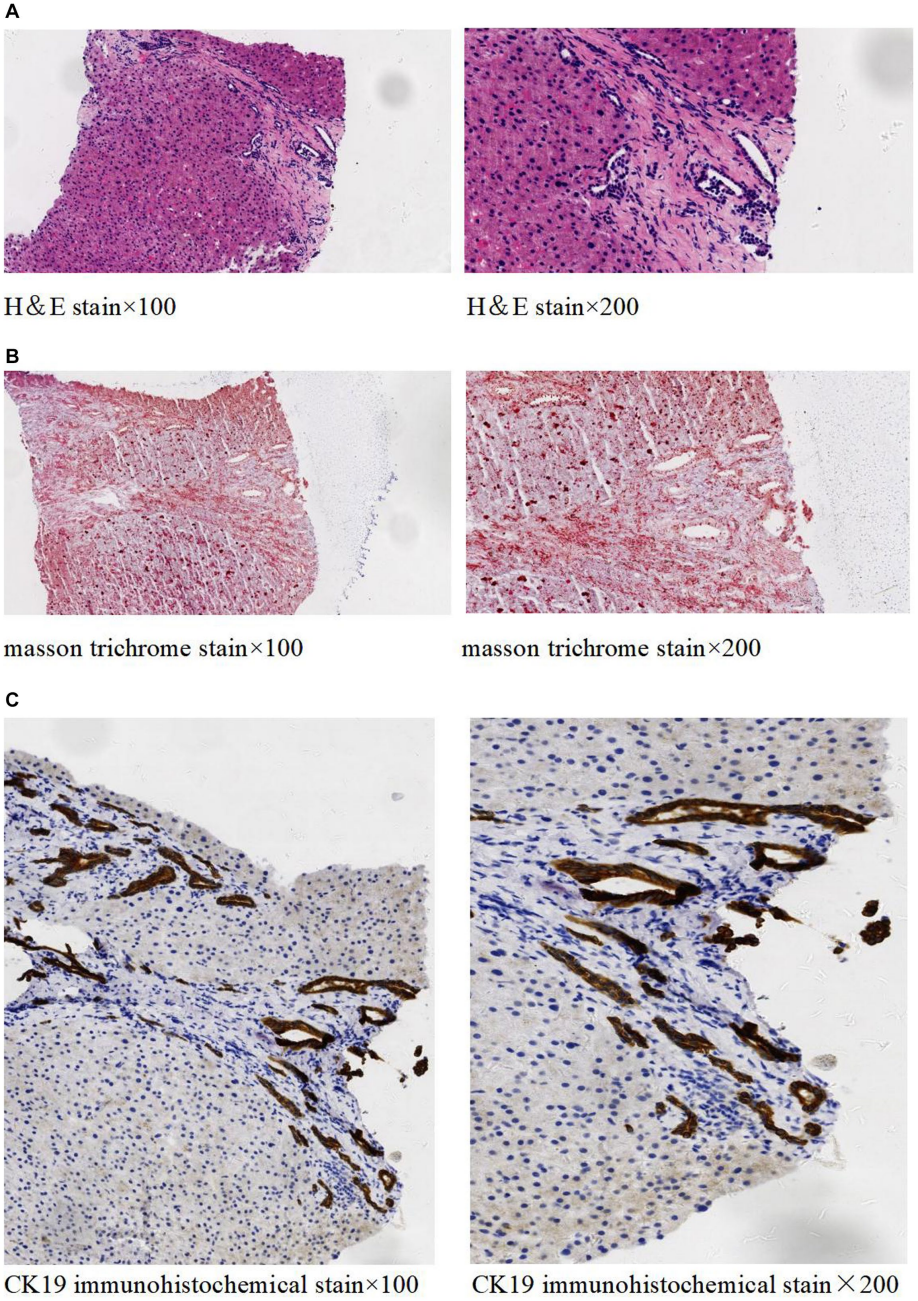
Histopathology of liver tissue. H&E staining **(A)** showed no significant lobular inflammation, nor signs of cholangitis, but there was portal tract enlargement with bridging fibrosis. Masson trichrome staining **(B)** demonstrated the enlargement of the portal area along with collagen fiber proliferation. Immunohistochemical staining of CK19 **(C)** revealed significant small bile duct proliferation in the portal areas.

The abdominal ultrasound showed signs of cirrhosis, splenomegaly, and portal vein dilation. An enhanced CT scan showed liver cirrhosis, splenomegaly, polycystic kidneys, a small volume of ascites, and portal hypertension with open collateral circulation (Figures 1A–C). Additionally, MRCP revealed dilation of the common and intrahepatic bile ducts, as well as wall thickening and edema of the gallbladder (Figure 1D). A liver biopsy (Figure 2) showed visible nodules without obvious intralobular inflammation, enlarged portal areas with bridging fibrosis, and a significantly increased number of small biliary ducts of various shapes. Histopathology confirmed CHF.

**Figure 3:**
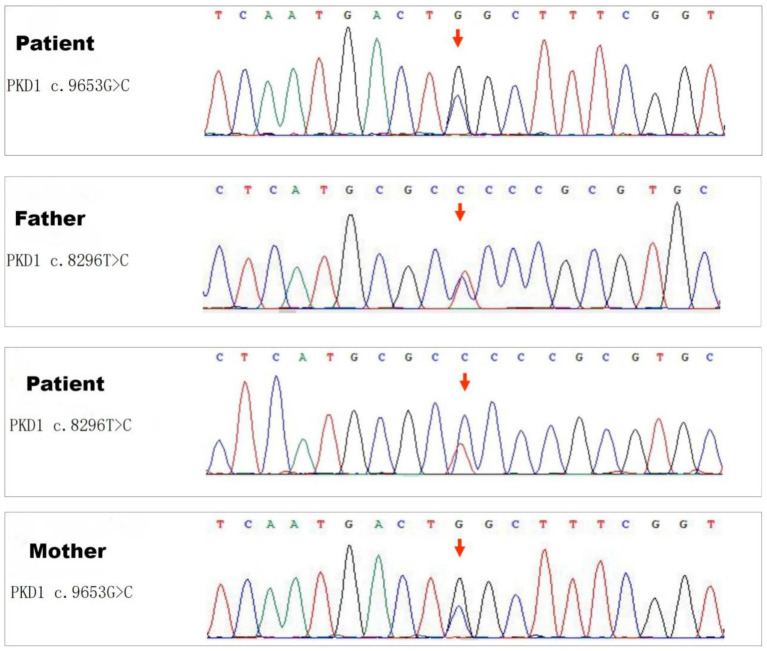
Sequencing results of the PKD1 gene. Genetic analysis revealed two distinct missense mutations in the PKD1 gene: one is a paternally inherited mutation c.8296 T > C (Ser2766Pro), and the other is a maternally inherited variant c.9653G > C (Trp3218Ser).

Genetic analysis revealed two distinct mutations in the PKD1 gene (Figure 3), determined to be biallelic mutations consisting of a paternally inherited mutation (c.8296 T > C) and a maternally inherited mutation (c.9653G > C). The former mutation results in an amino acid change from serine to proline at position 2,766, whereas the latter causes an amino acid change from tryptophan to serine at position 3,218.

Gastroscopy revealed severe esophageal and gastric variceal bleeding. Due to her economic status, the patient refused a liver transplant and instead received endoscopic variceal ligation and injection sclerotherapy. After treatment, the patient was discharged in improved condition and survived through the 18-month follow-up period. No variceal re-bleeding was observed during this time. A re-examination gastroscopy after 18 months showed a significant reduction in esophageal and gastric varices.

## Discussion and conclusion

Congenital hepatic fibrosis (CHF) is a rare hereditary fibrocystic liver disease with an incidence of about 1/10000 ~ 1/20000 (8). In previous literature reports, CHF has demonstrated a clear familial genetic tendency, and consanguineous marriage can lead to an increase in the prevalence of CHF (9). The pathogenesis of CHF is not fully understood, but the most widely accepted theory attributes it to ductal plate malformation (DPM), which leads to developmental abnormalities in the bile duct system. Proteins encoded by PKD genes (polycystin 1, polycystin 2, and fibrocystin) are known to localize to primary cilia, and the normal development of the portal vein system (10) and renal tubules (11) requires the intact ciliary signaling of PKD proteins. Mutations in PKD genes can cause ciliary dysfunction in biliary epithelial cells, leading to the onset of DPM (12). DPM is the underlying morphological abnormality in all fibrocystic diseases, resulting in an excessive number of embryonic bile ducts and abnormal portal vein branching, characterized by the presence of numerous embryonic-like bile duct structures in the porta hepatis (13). This immature bile duct system triggers inflammation and necrosis, promoting collagen deposition and the formation of fibrous tissue around the portal vein, resulting in the occurrence of CHF and related clinical symptoms such as recurrent cholangitis and complications associated with portal hypertension. The liver function of patients is usually normal or only mildly abnormal due to the absence of significant inflammatory response in hepatocytes.

Due to the nonspecific clinical manifestations of CHF, it is prone to being misdiagnosis or overlooked. The diagnosis of this disease primarily relies on imaging, pathological, and genetic examinations, with liver histopathology being the gold standard. Typical imaging features (14) include the absence of stenosis or occlusion of the portal vein at the porta hepatis, along with decreased, narrowed, or compressed intrahepatic portal vein branches. CT, Magnetic Resonance Imaging (MRI), and other examinations can reveal multiple dilated intrahepatic bile ducts and hepatosplenomegaly. The characteristic pathological findings include enlarged portal areas with bridging fibrosis, proliferating small bile ducts with diverse morphology within fibrous septa, and underdeveloped portal vein branches. In this study, we reported a case of a 31-year-old female patient who was admitted to our hospital with a chief complaint of hematemesis and melena for one day. She had no prior history of gastrointestinal bleeding. The findings of the patient’s examination are consistent with the characteristics of CHF. CHF is usually classified into four types, each with its distinct clinical features: the portal hypertension type, the cholangitis type, the mixed type, and the latent type (15). Given the patient’s clinical symptoms, imaging findings, and the results of the liver histopathological examination-which included portal hypertension and dilated extrahepatic and intrahepatic bile ducts-a diagnosis of portal hypertension type CHF was established.

In the medical literature, it’s well-established that patients with CHF often have concomitant kidney diseases. ARPKD is considered the most common association. Less frequently, there can also be concurrent cases of ADPKD, renal cystic dysplasia, or medullary cystic kidney disease (16). CHF is also linked to a variety of ciliopathies, such as Joubert syndrome (17), Senior-Loken syndrome (18), COACH syndrome (19), Meckel syndrome (20), and Bardet-Biedl syndrome (21). They typically manifest in infancy or early childhood with a spectrum of symptoms including ataxia, developmental delays, and intellectual disabilities, as well as various retinal defects and polydactyly, in addition to CHF. However, in the adult case at hand, the patient did not present with these clinical symptoms but solely with CHF and multiple bilateral renal cysts. Radiological examinations aligned the renal findings with polycystic kidney disease. We advanced our investigation using high-throughput sequencing technology for whole-exome sequencing, which identified two novel missense mutations in the PKD1 gene. It is known that pathogenic variants in PKD1 are responsible for about 85% of ADPKD cases (22). The mutations in PKD1 are diverse, including truncating, missense, splice site, deletions, and nonsense mutations. Although missense mutations sometimes do not alter protein function and may even confer beneficial effects, they most commonly lead to detrimental or lethal consequences.

These specific missense mutations have not been previously recorded in any public genetic variant databases (GnomAD, the 1,000 Genomes Project, or ExAC) or in specialized variant reference databases (ClinVar or Mastermind). However, at codon Trp3218, two missense alterations (Trp3218Arg and Trp3218Gly) and one nonsense mutation (Trp3218Ter) have been reported multiple times (23, 24). To our knowledge, missense mutations at codon Ser2766 have not been reported. Predictive *in silico* tools such as PolyPhen-2, M-CAP, MutationTaster, and SIFT indicate that these variants could be deleterious. The patient’s mother exhibited no clinical signs of kidney or liver disease, raising the possibility of incomplete penetrance, which could explain the transmission of the mutation without the manifestation of the disease. In contrast, the patient’s father showed clinical signs of hepatomegaly, splenomegaly, and multiple bilateral renal cysts, suggesting that the variant in Exon 23 c.8296 T > c is likely pathogenic. Regrettably, the patient’s brother declined genetic testing for personal reasons. Nevertheless, the familial pattern of the disease suggests an autosomal dominant mode of inheritance. After a thorough diagnostic evaluation, the patient was diagnosed with CHF alongside ADPKD.

Typically, CHF and portal hypertension are not commonly seen together with ADPKD (16). Instead, polycystic liver disease (PLD) represents the most frequent extrarenal manifestation of ADPKD (25, 26), typically not impairing liver function. By the age of 60, approximately 77% of ADPKD patients will have developed liver cysts. PLD is characterized by numerous fluid-filled cysts derived from the bile ducts. When a significant increase in liver volume occurs, it can lead to subjective discomfort due to mass effect (27). Misra A and colleagues described an elderly ADPKD patient with extensive liver and kidney cysts, where a liver biopsy showed that numerous liver cysts were compressing the portal and smaller veins, resulting in portal hypertension (28). In our unique case, the extrarenal manifestation of ADPKD manifested as CHF with accompanying portal hypertension, a relatively rare presentation.

In Table 1, we report the hepatic and renal characteristics of CHF patients with ADPKD as described in nine publications (5–7, 29–34) from 1985 to 2022. Table 1 includes 14 patients from 13 families, comprising 5 males and 9 females. The age of disease onset ranged from 2 to 33 years. Except for one patient (34), all had a definitive family history of ADPKD. Hepatic presentations were categorized as follows: three cases of latent type CHF without portal hypertension or cholangitis, nine cases of CHF with portal hypertension, and two cases of mixed type CHF featuring both portal hypertension and cholangitis. Regarding renal manifestations, one case of ADPKD progressed to end-stage renal disease (31), one patient experienced a decline in renal function during pregnancy necessitating peritoneal dialysis (33), while the remaining 12 patients maintained essentially normal renal function. Given the date range of the articles, including the 1980s and 1990s, genetic testing might not have been widely accessible. Therefore, during that time, six patients did not undergo genetic testing. Additionally, one 8-year-old child opted out of genetic testing for reasons not disclosed. In total, seven patients did not receive genetic testing for ADPKD. The other seven were genetically tested. The findings showed that five patients with CHF and ADPKD had mutations exclusively in the PKD1 gene (6, 7), including two missense mutations, two deletions, and one mutation of unspecified nature within the PKD1 gene. One individual (34) possessed a mutation in the ATP7B gene along with two mutations in the PKD1 gene. Lastly, one case (30) had genetic testing that, likely due to technological limitations of the time, only identified mutations associated with ADPKD onset without specifying whether they were in PKD1, PKD2, or another gene.

**Table 1:** Summary of characteristics of congenital liver fibrosis with ADPKD cases.

Case references	Age	Gender	Family history	CHF presentation	Kidney presentation	Other manifestations	Genetic findings	Treatment strategies
Lee and Paes (29)	7 years old	Female	The father has a history of ADPKD and hypertension	CHF with portal hypertension	Intravenous urography showed large rounded cysts with stretching and distortion of the calyces. Mildly abnormal serum creatinine.	Not mentioned	Genetic testing was not performed	Spleno-renal anastomosis, end-to-side porta-caval shunt, and endoscopic ligation were employed to treat recurrent ruptured esophagogastric variceal bleeding.
Cobben et al. (30)	29 years old (Family PK2)	Female	The mother had bilateral cystic kidneys, pancreatic cysts and an enlarged liver. One of her sisters was diagnosed with congenital hepatic fibrosis and bleeding esophageal varices. Intravenous pyelography revealed bilateral enlarged kidneys.	CHF with portal hypertension	Renal cyst. Normal serum creatinine.	Pancreatic cyst	DNA markers on chromosome 16 showed close linkage of these markers with the locus for ADPKD. A single cross over distal to the ADPKD locusoccurred among 15 meioses, 11 of which were informative for ADPKD.	The patient underwent splenectomy at the age of 8 due to splenomegaly.
	29 years old (Family PK67)	Male	The mother has bilateral cystic kidneys. His sister was found to have bilateral enlarged cystic kidneys and hepatic and splenic enlargement at birth.	CHF	Sonography revealed bilateral enlarged cystic kidneys. Normal serum creatinine.	An unexplained azoospermia	Genetic testing was not performed	Regular follow-up without treatment
	19 years old (Family PK11)	Male	The mother, two maternal aunts and the maternal grandmother are known to have ADPKD.	CHF with portal hypertension	Sonographic examination revealed cysts in bilateral enlarged kidneys. Normal serum creatinine.	Not mentioned	Genetic testing was not performed	Not mentioned
Matsuda et al. (31)	33 years old	Male	The father had ADPKD and polycystic liver	CHF	ADPKD progresses to the ESRD stage	Hypertension，proteinuria.	Genetic testing was not performed	The patient’s hypertension treatment failed. He died in a coma at the age of 33 due to a cerebral hemorrhage.
Lipschitz et al. (32)	19 years old (Splenomegaly presented at the age of 4)	Female	The mother proved to have PKD and hypertension; The maternal grandfather died for renal disease of unknown etiology at 46 years of age. Two maternal aunts, age 43 and 54 years, a 47-year-old uncle and a 31-year-old sister are known to have polycystic kidneys.	CHF with portal hypertension	Typical ultrasound findings of ADPKD but normal renal function	Not mentioned	Genetic testing was not performed	The patient was treated with sclerotherapy of esophageal varices using sodium tetradecyl sulfate; beta-receptor blockade therapy (propranolot) was simultaneously started.
Klinkert et al. (33)	32 years old (The initial onset of the disease was at the age of 14)	Female (primigravida)	She was a member of a family with ADPKD, linked with CHF	CHF with portal hypertension	Renal function slightly impaired; During the patient’s pregnancy, her renal function further deteriorated	moderate hypertension	Genetic testing was not performed	At age 14, the patient had splenorenal shunt placement and a splenectomy due to esophageal variceal bleeding from portal hypertension. During pregnancy, she received peritoneal dialysis for declining renal function.
Kanaheswari et al. (5)	8 years old	Female	The father was diagnosed with ADPKD	CHF with portal hypertension	Abdominal examination revealed a ballotable left kidney while an ultrasound of the abdomen revealed cystic lesions in the kidneys; normal renal function	Hypertension	Genetic testing was not performed	Not mentioned
O’Brien et al. (6)	4 years old 12 years old (Family1)	Male Female	They have a family history of ADPKD	CHF with portal hypertension	Kidney imaging showed multiple macrocysts in both kidneys consistent with ADPKD	Not mentioned	A deletion mutation in the PKD1 gene	The youngest of the children was started on propanolol and subsequently underwent distal splenorenal shunt placement.
	33 years old (Family2)	Female	The father and two children all exhibit typical renal manifestations of ADPKD.	CHF with portal hypertension and cholangitis.	Typical renal manifestations of ADPKD.	Not mentioned	A missense mutation in the PKD1 gene	The patient showed signs of splenomegaly at 6 months old. Throughout her childhood and adulthood, she underwent repeated banding for esophageal varices.
	36 years old (Family3)	Female	The mother died of renal complications of ADPKD	CHF	The kidneys contained multiple cysts	Not mentioned	A missense mutation in the PKD1 gene	Not mentioned
Jiang et al. (34)	25 years old (Case1)	Female	Not mentioned	CHF with portal hypertension and cholangitis	Polycystic kidneys	Not mentioned	Three heterozygous missense mutations (one in ATP7B and two in PKD1)	Three months prior to admission, the patient had a splenectomy to address her splenomegaly and associated thrombocytopenia. Financial constraints led her to forgo a liver transplant in favor of symptomatic antifibrotic care. During a two-year follow-up, she survived with recurrent ascites, partially managed by paracentesis and periodic albumin infusions.
Sila et al. (7)	8 years old (2-year-old onset)	Male	He has a family history of polycystic kidney disease	CHF with portal hypertension	Abdominal ultrasonography revealed the presence of numerous millimetric cysts on both kidneys.	Cranial magnetic resonance angiography and echocardiography did not reveal any other abnormality.	A heterozygous pathogenic variant was identified in the PKD1 gene.	Regular follow-up without treatment

For patients with CHF, there is currently no specific therapeutic method that can halt or reverse the disease’s progression. Treatment options include supportive care and management of complications related to CHF, such as antifibrotic drug therapy, anti-infection therapy for cholangitis, and interventions to manage portal hypertension (2). The patient in this case was admitted to the hospital due to severe hematemesis and melena. Gastroscopy revealed severe esophageal/gastric variceal hemorrhage as a result of portal hypertension. The patient did not exhibit symptoms of fever or abdominal pain associated with cholangitis. Consequently, symptomatic treatment for this patient could include endoscopic interventions, percutaneous transhepatic portosystemic shunt (TIPS), or surgical procedures to control portal hypertension and arrest the bleeding from the esophageal/gastric varices.

Endoscopic variceal ligation (EVL), often used with gastric variceal obturation (35), is a standard therapy for esophageal and gastric varices caused by portal hypertension, known for its minimal invasiveness and cost-effectiveness. Studies have confirmed its efficacy in controlling bleeding and improving survival (36). Transjugular intrahepatic portosystemic shunt (TIPS) is also a common management strategy for portal hypertension complications (37, 38), providing symptom relief and serving as a bridge to liver transplantation. However, it’s not suited for all due to risks like hepatic encephalopathy. Surgical options remain secondary to transplantation for medication-resistant portal hypertension, with procedures like laparoscopic distal splenorenal shunt offering positive short-term results in children with CHF (39). Liver transplantation emerges as the optimal treatment for patients with CHF in the terminal stage of liver disease (40, 41). In our reported case, the patient with decompensated liver disease faced financial barriers to transplantation. Consequently, the individual pursued EVL in conjunction with tissue adhesives and sclerotherapy, which led to satisfactory short-term results, with no complications observed during an 18-month monitoring period and notable variceal improvement upon subsequent evaluations.

In essence, this report introduces a unique instance of CHF concomitant with ADPKD in an adult female, enhancing the understanding of CHF’s clinical and radiographic presentations linked to ADPKD. This case highlights CHF’s association with ADPKD and underscores the importance of considering CHF in patients with unexplained liver cirrhosis, biliary abnormalities, and polycystic kidneys. Endoscopic management offers an effective alternative for bleeding control in CHF when transplantation is not immediately viable, though transplantation remains the best option for end-stage CHF.

## Data availability statement

The datasets presented in this article are not readily available because ethical/privacy restrictions. Requests to access the datasets should be directed to liuyingag@126.com.

## Ethics statement

The studies involving humans were approved by Tianjin third central hospital. The studies were conducted in accordance with the local legislation and institutional requirements. The participants provided their written informed consent to participate in this study. Written informed consent was obtained from the individual(s) for the publication of any potentially identifiable images or data included in this article.

## Author contributions

YL: Writing – original draft. PZ: Writing – review & editing. JT: Data curation.
